# Spanish psychometric properties of the moral distress scale—revised: a study in healthcare professionals treating COVID-19 patients

**DOI:** 10.1186/s12910-023-00911-2

**Published:** 2023-05-12

**Authors:** L Galiana, C Moreno-Mulet, A Carrero-Planells, C López-Deflory, P García-Pazo, M Nadal-Servera, N Sansó

**Affiliations:** 1 Department of Methodology for the Behavioral Sciences, University of Valencia, Valencia, Spain; 2 Department of Nursing and Physiotherapy, University of Balearic Islands, Valldemossa Road, Km 7,5., Palma, Balearic Islands 07122 Spain; 3 Balearic Islands Health Research Institute (IDISBA), Palma, 07120 Spain; 4 Balearic Islands Health System. Servei Balear de Salut (IB-Salut), Palma, Spain

**Keywords:** Moral distress Scale, Compassion fatigue, Validation study, Health personnel, Proqol 5, Psychometric property analysis, Spanish version

## Abstract

**Background:**

Moral distress appears when a healthcare professional is not able to carry out actions in accordance with their professional ethical standards. The Moral Distress Scale-Revised is the most widely used to assess levels of moral distress, but it is not validated in Spanish. The aim of the study is to validate the Spanish version of the Moral Distress Scale – utilised within a sample of Spanish healthcare professionals treating COVID–19 patients.

**Methods:**

The original (english) and the portuguese and french versions of the scale were translated into spanish by native or bilingual researchers and reviewed by an academic expert in ethics and moral philosophy as well as by a clinical expert. Research design: Descriptive cross-sectional study carried out using a self-reporting online survey. The data was collected between June- November 2020. A total of 661 professionals responded to the survey (N = 2873). Participants: healthcare professionals with more than two weeks of experience treating COVID–19 patients at the end of their life and working in the public sector of the Balearic Islands Health Service (Spain). Analyses included descriptive statistics, competitive confirmatory factor analysis, evidence on criterion-related validity and estimates of reliability. The study was approved by the Research Ethics Committee at the University of Balearic Islands.

**Results:**

An unidimensional model in which a general factor of moral distress explained by 11 items of the Spanish version of the MDS–R scale was an adequate representation of the data: *χ*^*2*^(44) = 113.492 (*p* 0.001); Comparative Fit Index = 0.965; Root Mean Square Error of Approximation = 0.079[0.062,0.097]; and Standarized Root Mean-Square = 0.037. Evidence of reliability was excellent: Cronbach’s alpha = 0.886 and McDonald’s omega = 0.910. Moral distress was related to discipline, with nurses having statistically significant higher levels than physicians. Additionally, moral distress successfully predicted professional quality of life, with higher levels of moral distress being related to poorer quality of life.

**Conclusions:**

The Spanish version of Moral Distress Scale–Revised can be used as a reliable and valid measurement tool for the evaluation of moral distress experienced by health professionals. This tool will be highly useful for managers and applicable to a variety of healthcare professionals and settings.

**Supplementary Information:**

The online version contains supplementary material available at 10.1186/s12910-023-00911-2.

## Background

During the COVID-19 pandemic, healthcare professionals (HCP) were faced with a multitude of new professional challenges: the lack of access to adequate protective equipment [1–4]; feeling inadequately supported; exhaustion from wearing personal protective equipment for entire working shifts [5]; prolonged working hours and unexpected changes in the type of work demanded of them [6]; limited knowledge on updated information as well as constantly changing guidelines [5, 7]; and uncertainty surrounding the efficacy of disease containment [1, 8]. These stressors were exacerbated for those HCP who cared for COVID–19 patients who died alone [9], with no relatives or opportunity for farewells. This situation produced incredible suffering and stress, not only for these patients and their families, but also for the HCP, who experienced anxiety, depression, stress, burnout, and moral distress [2, 4–6, 9–11]. Some of these situations derived from the moral conflicts they had to face [12, 13].

## Moral distress

Moral distress was first defined by Jameton [14] as the distress that arises when “one knows the right thing to do, but institutional constraints make it nearly impossible to pursue the right course of action”. Similarly, Wilkinson [15] defined moral distress as “the psychological disequilibrium and negative feeling state experienced when a person makes a moral decision but does not follow through by performing the moral behavior indicated by that decision”. These definitions demonstrate that the foundation of moral distress experienced by HCP results from them not being able to carry out actions in accordance with their professional ethical standards [16, 17].

The most common triggers for moral distress, some of them similar to those that cause burnout [8, 18], are: working alongside professionals with inadequate capabilities or training, which can result in unsafe situations; identifying patient suffering caused by a lack of continuity of care or having to carry out unnecessary interventions or those that are perceived to cause discomfort; or having to adhere to institutional policy that interferes with patient care [19, 20]. Of the factors that cause burnout that can also cause moral distress, we can highlight those that are related to professional autonomy [21], especially the autonomy of the nurses, stressors related to their work environment, the type of assistance they provide, as well as the economic and social context [8, 18].Consequently, it is necessary to identify which are the relevant factors in the detection of moral stress, as is being done in other similar phenomena such as burnout [18, 22], and it is also necessary to have tools to be able to identify the characteristics of the professionals, health institutions and health systems.

In this sense, it should be explained that the health system in Spain is public and with universal coverage, therefore it provides free care to all people regardless of their economic or administrative situation. So this is not a factor that generates moral stress like in other countries, for example the United States [8, 23]. However, during the pandemic the factors that caused the most stress were: the lack of available material and human resources, the increase in workloads in almost all settings [8], and the emotional impact suffered during health care in the pandemic [2, 24] has been identified as one of the core factors that reduces professionals’ well-being and quality of patient care [25]. Some of the unfavorable outcomes that have been highlighted amongst the literature include anxiety, frustration, social isolation, sadness, helplessness, feeling of guilt, depression, negative changes in self-image and spirituality, headaches, substance abuse, or digestive disorders [15, 26–30]. This clearly also increases the risk of staff turnover, early retirement, and long-term absences from work, resulting in high levels of burnout and lack of job satisfaction and professional quality of life) [8, 18, 22, 31–36]. As it is pointed out by the literature, it must be noted that professional quality of life increases professionals’ well-being [37, 38], thus resulting in lower incidence of medical errors, sick leaves and absenteeism [39], as well as better quality of care [36]. Indeed, moral distress was negatively related to age [40], work experience [41], effective communication [42, 43] and compassion satisfaction [31], whereas it showed a positive relation with burnout and compassion fatigue [38]. More recently, Malliarou et al. [44] have delved into these relationships, again with evidence pointing a negative relation between moral distress and professional quality of life (higher levels of burnout and compassion fatigue were related to higher scores in moral distress).

Despite the fact that moral stress can affect all health professionals, from the review of the bibliography it is concluded that nurses are particularly susceptible to suffering the highest levels of moral stress. [20]. Consequently, the quality of nursing care wane significantly in the face of moral distress [19, 45].

Some authors affirm that nurses, and especially female nurses [46], they are more sensitive to suffering moral distress due to the strong identity they have established around caring for the population and, on the other hand, a very close relationship with users that places them in a situation of strong commitment and responsibility towards them. Among the care environments where the moral stress of nurses has been studied, the intensive care units stand out [47].

Abassi [48] shows evidence that there are few studies aimed at analyzing the moral stress of doctors and these professionals can also suffer moral anguish due to different factors such as the inability to provide care to users who do not have economic resources, inefficiency in management, lack of resources, the lack of time to serve users and other organizational aspects. In general, the few studies on moral stress in doctors are aimed at comparing, in the same situations, the results obtained with the levels of moral stress in nurses. In general, doctors present moral distress, although they do so to a lesser degree than nurses. [47, 49].

In consequence, the complexity of the phenomenon requires both a quantitative and qualitative approach in order to fully understand it, however, the use of research instruments such as scales and questionnaires allows to quickly identify the presence of professional moral distress.

## Moral distress scales

Over the past twenty years of research on moral distress, several scales and questionnaires have been developed. Two recent systematic reviews identified up to eight different instruments [25, 50]: (a) the Ethical Stress Scale (EES) [51], which was one of the first to be developed and aimed to explore the relationships among exposure to ethical issues, moral reasoning, coping style and ethical stress; (b) the Moral Distress Scale (MDS) [52], and its accompanying revised models such as the Moral Distress Scale–Revised (MDS–R) [53, 54]; (c) the Sweden Stress Conscience Questionnaire (SCQ) [55], assessing internal demands and external demands and restrictions; (d) the Moral Distress Questionnaire (MDQ) [56], a culturally-sensitive questionnaire aiming to assess the moral distress among nurses employed in a variety of work settings; (e) the Moral Distress Thermometer (MDT) [57], an instrument that measures real-time moral distress in hospital nurses; (f) the Moral Distress Intensity [58], a tool which assesses the intensity of moral distress among nurses; (g) the Canada Moral Distress in Dementia Care Survey (MDDCS) [59], which assesses the triggers of moral distress, the potential effects of moral distress on the respondent, job satisfaction, and strategies that may mitigate moral distress; and (h) the Measure of Moral Distress for HCP (MMD-HP) [60], which includes twenty-seven items and is applicable to healthcare practitioners in critical, acute, or long-term care settings. Among all of these tools, the MDS and its revised version, the MDS–R, are undoubtedly the most widely used. This can be exemplified by a review carried out by Giannetta et al. [25], which showed that 79 out of 88 studies carried out included Corley’s instruments on moral distress.

The MDS was developed by Corley et al. [52] from interview data and a comprehensive literature review, to assess moral distress in intensive care nurses, and was originally composed of thirty-two items. In a second version, Corley et al. [52] added six more items that inquired over pain management and the management of care and personnel. In 2007, Hamric et al. [53] condensed the MDS from 38 to 19 items, and used these items to ask about both the frequency of moral distress and its intensity. In 2012, Hamric et al. [53] added two more items to the MDS, modifying it so that it could be applied to all HCP working in intensive and acute clinical settings. This latest version has been used in many clinical settings, and has been validated in different languages, including Brazilian-Portuguese [61, 62], Farsi [48], German [63], Swedish [64, 65], Iranian [34], Greek [66], Turkish [67], Italian [68–70], and Persian [71, 72]. These studies have found evidence of a diverse internal structure, varying from one to seven factors, although the initial studies by Corley et al. [52, 73] and Hamric et al. [53, 54] assumed a one-factor structure.

## Purpose

Thus, as explained by different authors [65, 72]. MDS-R has demonstrated adequate reliability and construct validity. The items are phrased as statements and for each statement, the respondents are asked to indicate, on a 0–4 Likert scale, both the frequency (how often the situation arises) and the level of disturbance (intensity) when the situation arises. The respondents are also asked to indicate intensity, even if they have not experienced a situation. However, to date, there have been no findings of a Spanish validation of the MDS–R scale, nor of the other scales mentioned before, despite being the second most spoken language in the world and the relevance of the phenomenon. Based on this identified need, this study has the intention to provide a validated scale that can be used both in the clinical and research settings in Spain (and other Spanish-speaking countries) in order to assess moral distress in HCP.

## Methods

The aim of our study was to validate the Spanish version of the Moral Distress Scale – Revised within a sample of Spanish HCP caring for COVID–19 patients. In addition, this research also aimed to study the influence of other variables of moral distress within the study sample, and the impact of moral distress on professional quality of life.

### Design and procedure

This was an instrumental study that was carried out using a self-report online survey assessing moral distress. The survey was created using the ‘SurveyMonkey’ platform.

### Setting and participants

The study was carried out on HCP caring for COVID–19 patients and working in the Balearic Islands (Spain) public hospitals. The access to the sample began by contacting by email with Balearic Health Service and public hospital managers for the presentation of the project, who sent two corporative emails to all the professionals working at the health services (N = 2873). Then, access to the sample finished by contacting by email with head physicians and middle nurse managers of the COVID–19 services or units, who sent corporative emails or mobile messages to all the professionals working in their areas. These emails or messages contained the explanation of the study and the access to the online survey. The response rate was 23% (n = 661). Data collection started in June 2020 and finished in November 2020.

The inclusion criteria for participants were (a) professionals working at the time of the survey; (b) having more than two weeks of work experience caring for COVID-19 patients (this time was considered sufficient to be able to know the impact of care in a pandemic); and (c) caring for COVID-19 patients at the end of their life. Inclusion criteria responded to the fact that the study is part of a larger study in which it was intended to assess the effect of the pandemic on professionals. Among other study variables, the project intended to measure the effect on moral distress. As this tool was not validated in Spanish, it was validated in order to provide a Spanish version to the scientific community.

A minimum sample size was established at 190 participants, in accordance with Wolf et al. [74], who showed that a one-factor solution with four indicators would require a sample size of 190 participants with lowest factor loadings of 0.50. Taking this into account, more indicators require smaller samples [73]. The Moral Distress Scale – Revised was initially composed and utilised with 21 items, n = 190 was the most conservative choice. The survey was not limited to this sample size, but the maximum possible participation was sought after for the sake of better representation.

### Variables

Together with sociodemographic characteristics (age and sex) and workplace characteristics, such as professional discipline, hospital unit, and type of contract, information on moral distress and professional quality of life was gathered.

To assess moral distress, the Spanish version of the Moral Distress Scale – Revised was used. The original English version of the scale was translated into Spanish using the ‘backward and forward translation process’ [75]. The process of translation was preceded by a literature review to assess conceptual and item equivalence from the original to the targeted context [76] .The scale was first translated from the source to the target language (Spanish) by one of the authors of this article who are native Spanish and English speakers. Another native French-Spanish researcher translated the French version [77]. The synthesis of these two translated versions gone through a triangulation process with the Portuguese versions [61, 62] of the scale with no discrepancies found between them. It should be pointed out that despite the contextual differences between these countries, in other validation studies of instruments to assess burnoutt, such as the one of Manzano y Ayala [18], in which HCP from different European countries have participated, results with a high level of consensus have been achieved. A committee approach was then used to achieve consensus among the two versions of the Spanish translated scale. Both versions of the scale were reviewed by an academic expert in ethics and moral philosophy as well as by a clinical expert in fatigue compassion, burnout, and professional quality of life. The selected Spanish scale was then translated back into the source language (English) by a native Spanish and English speaker. No change in meaning comparing the back-translated version of the scale and the original one was found. The Spanish version of the Moral Distress Scale – Revised can be seen in Annex 1.

#### Annex 1. The spanish version of the moral distress scale – revised

In order to evaluate professional quality of life, the validated Spanish version of the Professional Quality of Life Scale (Short-ProQOL) [37, 78] was used. The ProQOL comprises of three subscales: compassion satisfaction, compassion fatigue, and burnout [79]. Each dimension is represented in the scale by three items that are scored using a 5-point Likert scale (ranging from 1 ‘never’ to 5 ‘very often’). Examples of items are “*I like my work as a helper*” for compassion satisfaction, “*I think that I might have been affected by the traumatic stress of those I help*” for compassion fatigue, and “*I feel worn out because of my work as a helper*” for burnout. The scores for each dimension are calculated as the sum of the three items and therefore range from 3 to 15. Reliability estimates in this study were 0.773 for compassion satisfaction, 0.769 for compassion fatigue, and 0.767 for burnout.

### Statistical analyses

The internal structure of the scale was assessed via confirmatory factor analysis (CFA). The a priori model for the questionnaire structure was based on theoretical reasoning [52–54] which resulted in a one-factor structure. Therefore, a CFA in which a general factor of moral distress explained the 21 items of the Moral Distress Scale – Revised was hypothesized and tested. This first structure did not adequately fit the data, so a second CFA was estimated and tested, this time using the best indicators (the items with higher factor loadings). Items with factor loadings higher of 0.60 (*λ* > 0.60) and best homogeneity (correlation item-test > 0.50) were used. The CFAs were estimated using Weighted Least Square Mean and Variance adjusted estimator (WLSMV), as recommended for ordinal and non-normal data [80].

In order to assess model fit, several criteria were used: (a) the chi-square statistic; (b) the comparative fit index (CFI); (c) the root mean squared error of approximation (RMSEA); and (d) the standardized root mean squared residuals (SRMR). A CFI above 0.90 (or, better, exceeding 0.95) and an SRMR or RMSEA below 0.08 (or better, below 0.05) indicated a good fit [81].

In addition, analyses included internal consistency checks for the included items, such as the items’ homogeneity and alpha if item was deleted as well as estimates of internal consistency for the scale (Cronbach’s alpha and McDonald’s omega).

Evidence for validity was based on the relationships between other variables, relating moral distress with sociodemographic characteristics, workplace characteristics, and professional quality of life. To relate moral distress scores with age and years of experience, Pearson correlations were used. In order to study moral distress differences across sex, disciplines, units, and contract type, several analyses of variance (ANOVAs) were calculated. Regarding the relationships between moral distress and the dimensions of professional quality of life, we studied them, first, with Pearson correlations. In a second step, and as relationships between moral distress and professional quality of life have been previously stated (i.e., Austin et al. [31]), we hypothesized and tested a structural equation model, in which a factor formed by the 11 items of the Spanish version of the Moral Distress Scale – Revised predicted a factor of professional quality of life, formed by the three dimensions of the Short ProQOL. The model was based on Austin et al. (2017) and Malliarou et al.’s [44] results, and therefore it posited an impact of moral distress on professional quality of life, which was expected to be negative. In order to evaluate the model’s fit, criteria stated above were used.

Analyses were performed using IBM SPSS Statistics for Windows, version 26.0 [82] and Mplus, version 8.7 [83].

## Results

### Participants’ description

The total sample was composed by 299 professionals. Mean age was 38.77 (SD = 9.90). 85.3% were women and 61.9% were nurses. Participants had been working as HCP for an average of 12.73 years (SD = 8.82). Descriptive statistics of the sample can be consulted in Table 1.

**Table 1:** Sample descriptive statistics

Variable/groups	n	%
Sex		
Women	255	85.3
Men	44	14.7
Missing	0	0.0
Discipline		
Physicians	23	7.7
Nurses	185	61.9
Nursing assistants	85	28.4
Others	5	1.7
Missing	1	0.3
Unit		
COVID-19 Hospitalization Unit	85	28.4
Intensive Care Unit	110	36.8
Emergency Unit	47	15.7
Others hospitalization units	56	18.7
Missing	1	0.3
Contract		
Permanent contract	102	34.1
Interim contract	58	19.4
Temporary contract	137	45.8
Missing	2	0.7

### Evidence of the internal structure of the Spanish version of the moral distress scale – revised

The a priori one-factor model, testing the structure of the Spanish version of the Moral Distress Scale – Revised, showed an inadequate fit to the data: *χ*^*2*^(189) = 576.001 (*p* < .001); CFI = 0.889; RMSEA = 0.090[0.082,0.099]; and SRMR = 0.063. In order to estimate a second model, those items with the higher factor loadings (*λ* > 0.60) and homogeneity estimates (correlation item-test > 0.50) were retained.

**Fig. 1:**
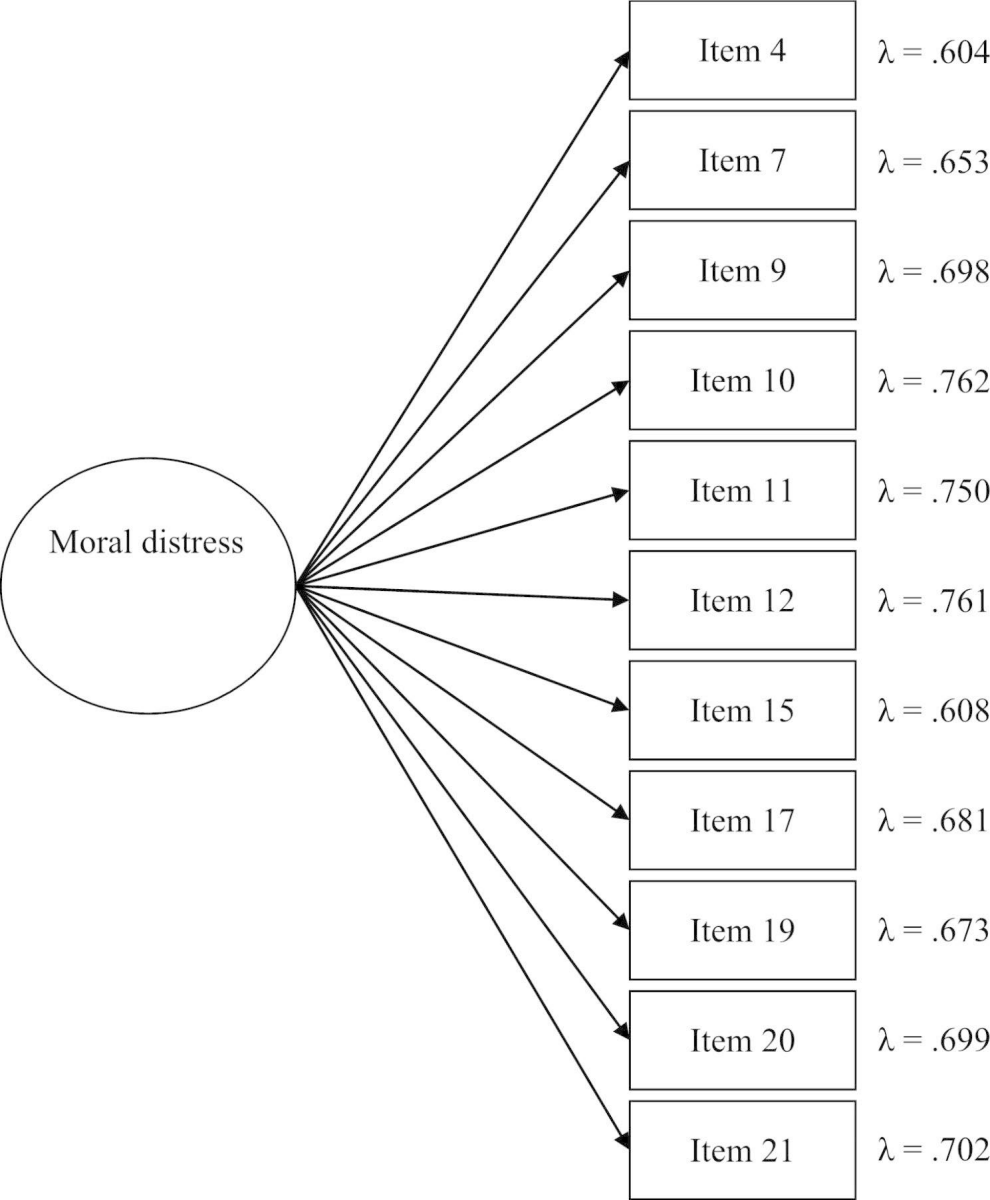
Analytical results of the Spanish version of the Moral Distress Scale – Revised Notes: All factor loadings were statistically significant (*p* < .001). For the sake of clarity, standard errors are not shown

Therefore, the items with the best psychometric properties in the Spanish version were used and a second CFA was tested. This time, only 11 items from the original Moral Distress Scale – Revised were used: items 4, 7, 9, 10, 11, 12, 15, 17, 19, 20, and 21. This second model showed excellent overall fit: *χ*^*2*^(44) = 113.492 (*p* < .001); CFI = 0.965; RMSEA = 0.079[0.062,0.097]; and SRMR = 0.037. As regards the analytical fit, this was also excellent. Items 10 (“*Be required to care for patients I don’t feel qualified to care for*”) and 12 (“*Provide care that does not relieve the patient’s suffering because the physician fears that increasing the dose of pain medication will cause death.*”) showed the higher factor loadings, whereas items 4 (“*Initiate extensive life-saving actions when I think they only prolong death*”) and 15 (“*Take no action about an observed ethical issue because the involved staff member or someone in a position of authority requested that I do nothing*”) showed the lowest ones. Details can be consulted in Fig. 1.

### Evidence of the reliability of the Spanish version of the moral distress scale – revised

Evidence for the reliability of the Spanish version of the Moral Distress Scale – Revised was excellent (Cronbach’s alpha = 0.886 and McDonald’s omega = 0.910). Items’ reliability estimates were also adequate. Correlations between items and the rest of the scale were also adequate (items’ homogeneity), ranging from 0.521, for item 4 (“*Initiate extensive life-saving actions when I think they only prolong death*”), to 0.674 for item 12 (“*Provide care that does not relieve the patient’s suffering because the physician fears that increasing the dose of pain medication will cause death*”). Additionally, when items were removed from the scale, alpha decreased in all the cases. More information on reliability estimates, together with items’ descriptive statistics, can be consulted in Table 2.

**Table 2:** Descriptive statistics and reliability estimates for the items of the Spanish version of the Moral Distress Scale – Revised

Item num.	M	SD	Item homogeneity	Alpha if item deleted
4	2.517	1.205	0.521	0.881
7	2.699	1.367	0.573	0.879
9	2.486	1.205	0.620	0.875
10	2.063	1.095	0.664	0.873
11	2.046	1.174	0.643	0.874
12	2.456	1.305	0.674	0.872
15	1.926	1.013	0.528	0.881
17	2.817	1.189	0.606	0.876
19	2.345	1.239	0.585	0.877
20	2.242	1.120	0.611	0.876
21	2.979	1.243	0.619	0.875

Notes: M = mean; SD = standard deviation

### Evidence of validity based on the relations with other variables of the Spanish version of the moral distress scale – revised

First of all, moral distress was linked to participants’ socio-demographic characteristics. The Pearson correlation that related moral distress with age resulted as non-statistically significant (*r* = − .114, *p* = .073), as the analysis of variance studying differences by sex (*F*(1,249) = 1.981, *p* = .160, *η*^*2*^ = 0.008) (see women and men’s means in Table 3).

**Table 3:** Descriptive statistics of moral distress across groups of sex, disciplines, unit and type of contract, and *p* value for the analyses of variance

Variable/groups		Moral distress		*p*
		M	SD	
Sex	Women	2.47	0.86	0.160
	Men	2.27	0.56	
Discipline	Physicians	2.03	0.48	0.005
	Nurses	2.56	0.78	
	Nursing assistants	2.29	0.97	
Unit	COVID-19 Hospitalization Unit	2.33	0.87	0.178
	Intensive Care Unit	2.52	0.80	
	Emergency Unit	2.61	0.88	
	Others	2.31	0.71	
Contract	Permanent contract	2.42	0.88	0.724
	Interim contract	2.37	0.68	
	Temporary contract	2.48	0.84	

Notes: *p* value correspond to analyses of variance

With regards to the workplace characteristics, the relation between moral distress and years of experience was first studied. The Pearson correlation yielded a not statistically significant result (*r* = − .091, *p* = .152). Then, moral distress was studied across all disciplines, units, and contract types. For the first analysis of variance, in which we studied means of moral distress across disciplines, one category, that of ‘others’, with only 5 participants, was re-coded into the missing values. The resulting ANOVA pointed statistically significant differences: *F*(2,244) = 5.405, *p* = .005, *η*^*2*^ = 0.042. When post hoc comparisons were performed, they pointed to lower levels of moral distress for physicians when compared to nurses (*p* < .001) (see Table 3). A second ANOVA studied the differences across units, with a non-statistically significant result: *F*(3,246) = 1.651, *p* = .178, *η*^*2*^ = 0.020. Similarly, the third ANOVA, where participants were grouped by type of contract, showed no statistically significant differences in moral distress: *F*(2,246) = 0.323, *p* = .724, *η*^*2*^ = 0.003. Indeed, as it is shown in Table 3, means were almost identical across groups.

Finally, moral distress showed a marginally significant negative correlation with compassion satisfaction (*r* = − .123, *p* = .054) and positive relations with compassion fatigue (*r* = .197, *p* < .001) and burnout (*r* = .279, *p* = .001). As the relationships between moral distress and professional quality of life have been previously stated, we used the Short-ProQOL [78, 84] to gather evidence for the Spanish version of the Moral Distress Scale – Revised. We tested a structural equation model, in which moral distress, with the added 11 items for the Spanish version of the Moral Distress Scale – Revised, predicted for the factor of professional quality of life, made up of the three dimensions of the Short ProQOL.

**Fig. 2:**
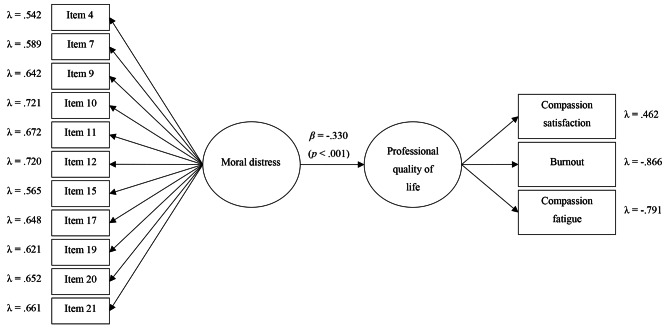
Analytical results of the model predicting professional quality of life Notes: All factor loadings were statistically significant (*p* < .001). For the sake of clarity, standard errors are not shown

The model showed an excellent overall fit: *χ*^*2*^(76) = 126.077 (*p* < .001); CFI = 0.958; RMSEA = 0.049[0.033,0.063]; and SRMR = 0.045. With regards to the measurement of the model, factor loadings for the Spanish version of the Moral Distress Scale – Revised were adequate, ranging from 0.542 to 0.721. The dimensions of compassion satisfaction, burnout, and compassion fatigue also did adequately load in the professional quality of life factor (*λ* = 0.462, *λ* = − 0.866, and *λ* = − 0.791, respectively). As to the predictive part, moral distress negatively predicted professional quality of life, with a statistically significant relationship (*β* = − 0.330, *p* < .001), explaining more than 10% of its variance (*R*^*2*^ = 0.109, *p* = .017). More details can be consulted in Fig. 2.

## Discussion

The aim of the study was to validate the Spanish version of the Moral Distress Scale – Revised in a sample of Spanish HCP caring for COVID–19 patients, as well as to test the structures previously identified in the literature, by using confirmatory factor analysis. To achieve this objective, the study was carried out on a sample of 299 HCP.

When the Moral Distress Scale – Revised was translated and tested in our sample, results of the CFA were poor. According to previous studies on MDS–R validation [53, 68, 70], we decided to retain only the best fitting items. The new model, with only 11 items, showed an excellent overall fit. The Spanish version of the Moral Distress Scale – Revised was represented by a one-factor structure, similar to the original approach [53, 54, 73], with all items being explained by a single factor of moral distress. That is, items from the Spanish version of the MDS–R represented behaviours of moral distress. A good example of conscious misconduct, that would surely have a great moral impact on the professional who carries it out was the item: “*Initiate extensive life-saving actions when I think they only prolong death”*. The Spanish version of the MDS–R showed excellent reliability, as well as items when individually studied, like in other studies that have validated the scale in other languages [61, 62, 65].

When analyzing moral distress and its relation to demographic characteristics, no relationship with age or sex was detected. This is not reflected in the results of other studies, such as those of Abdolmaleki et al. [21], Borhani et al. [40] or Babamohamadi et al. [41]. These authors found that age had a negative and significant correlation with the frequency and intensity of moral distress, in particular, that older professionals demonstrated lower levels of moral distress. In our study, no relationship with age was found, neither with years of experience [40, 41]. It is worth noting that also years of experience are expected to be a protective factor against moral distress, as older professionals are believed to be the most experienced and therefore able to develop coping skills to manage uncomfortable situations. A possible explanation for why age did not play a protective role in our sample is due to the pandemic situation: even the older professionals were not prepared to face a completely new and unexpected clinical situation. The biggest threat to morality for HCP were the scarce resources and their unequal distribution [4, 7], the lack of adequate protective equipment [4, 42, 43], and the restrictions on visitation rights, especially with regards to dying patients [4, 12].

Some studies found statistically significant differences in the moral distress experienced between the sexes, with a greater presence of moral distress in women, exemplified by Babamohamadi et al. [41] and O’Connell [46]. Other studies however are more in line with our own, which showed no differences [21, 67]. The absence of differences could be due to the global moral distress produced by the pandemic, which has been noted above. It must be pointed out however, that the sample included only 44 male participants. Although this study shows that women have higher scores in moral distress, this difference is not statistically significant. Usually when such differences are found, authors attribute them to women’s greater sensitivity and empathy [85]. This is, however, a topic to be further explored in future studies, as results here, as well as surrounding literature, are not clear.

When workplace characteristics were analyzed, no relationship of moral distress with years of experience was found, as stated above, neither differences on moral distress were found when comparing units or contract types [41]. However, a significant relationship was found regarding discipline. Nurses in particular, obtained higher levels of moral distress when compared to physicians, something which is in line with results obtained in earlier studies [9, 13, 31, 47, 49, 53]. As previously discussed in the literature, nurses are more likely to experience moral stressors, such as the lack of involvement in decision-making processes, little respect for nurses’ autonomy, the witnessing of unethical behavior by colleagues and working alongside colleagues in unsafe working conditions [71, 72]. In addition, it has been suggested that nurses can be morally injured if they perceive the unavailability of medical staff or an incongruence in the values of their organization [69, 71]. Consequently, when compared with physicians, nurses report higher levels of moral distress [9, 13, 31, 47, 49, 53].

Last but not least, our results have confirmed that moral distress acts as a predictor of professional quality of life, in line with findings from previous studies [31]. Considering moral distress consequences in HCP [26–30] and in quality of care [36–39], it is imperative to assess, and when possible prevent, the occurrence of moral distress in HCP [9].

### Implications

Moral distress is to be considered as a threat to HCP’ well-being and professional quality of life and, consequently, as a threat to quality of care in healthcare organizations. Spanish version of Moral Distress Scale–Revised validation fills the gap around the lack of validated scales for the assessment of moral distress in the Spanish healthcare context. The availability of this validated scale leads to practical implications at different levels.

Recognition of the prevalence and main causes of moral distress among HCP would allow managers to systematically and continuously monitor them. Therefore, organizational strategies for prevention and early correction of issues compromising professionals’ moral integrity could be implemented in clinical settings. Likewise, this metric information would constitute a strong argument for healthcare policymakers to position HCP’ professional quality of life and well-being as a priority, since quality of care depends on it.

Studies addressing moral distress assessed by means of this validated scale in the Spanish context would generate a body of knowledge not only on the relationship between moral distress and some sociodemographic variables (such as age, sex or work experience), but also on the comparison of the prevalence and causes of moral distress among different HCP, different clinical settings and different healthcare conditions. Knowledge generation in this regard could also be useful in educational settings to design training plans aimed at preparing students in health sciences to face the circumstances most likely to generate moral distress.

### Limitations

Due to the cross-sectional nature of this study, there is a lack of evidence for reliability with regards to the MDS–R test-retest. An additional limitation is the small sample size of male participants compared to females. However, regarding the feminization of the sample, it has to be kept in mind that this is a characteristic of the Spanish healthcare population, and so, in this sense the sample is still representative of the population. Another constraint is the incidental sampling method, which could affect the representativeness of our results. Also, and as it occurs with most of survey studies, nonresponse bias could not be assessed. In this sense, we observed a higher nonresponse bias in physicians, who were underrepresented in the sample, taking into account the healthcare population of the Balearic Islands. The number of professionals in each discipline was provided by the Care Department of the Health System. Therefore, results may be interpreted with caution. Finally, the relationship between moral distress and professional quality of life was not controlled for other variables, such as the effect of work variables (i.e., workload or work stress) or inner resources (i.e., self-care or self-compassion). Considering the multidimensionality of the construct of professional quality of life, and the fact that it has been linked to a plethora of variables, the role that moral distress plays within a larger set of variables should be further investigated.

In order to improve future studies, MDS-R longitudinal investigations should aim to include larger samples obtained via randomized sampling, therefore offering evidence of the stability of internal structure in bigger, randomized samples. Furthermore, studies including other health care environments and professionals would be welcomed.

## Conclusions

The Spanish version of Moral Distress Scale–Revised can be used as a reliable and valid measurement tool for the evaluation of moral distress experienced by HCP caring for COVID-19 patients in Spain. The present study confirms that nurses are at the highest risk from suffering moral distress, as well as the negative consequences this has on their professional quality of life.

Since the tool presented here is a Spanish revised version of an instrument derived by Corley (the Moral Distress Scale- Revised), which in itself is considered to be the most useful and appropriate tool for clinical and research settings, we suggest that this version will be highly useful for managers and applicable to a variety of HCP and settings.

Moral distress has been proven to be a threat to both the care of professionals, with an increase the risk of staff turnover and early retirement, and a decrease of professional quality of life, and to the care of patients. It seems clear then that Healthcare Systems must keep their professionals safe from moral distress. In order to do so, they must necessarily begin by establishing the prevalence and causes of the problem, something this newly revised tool can help achieve.

## Electronic supplementary material

Below is the link to the electronic supplementary material.

Supplementary Material 1: The Spanish version of the Moral Distress Scale

## Data Availability

The datasets generated and/or analysed during the current study are not publicly available due to privacy and confidentiality reasons, but are available from the corresponding author on reasonable request.

## List of Abbreviations

ANOVA: Analysis of variance.standardized
CFA: Confirmatory factor analysis
CFI: Comparative fit index
EES: Ethical Stress Scale
HCP: Healthcare professionals
MDDCS: Canada Moral Distress in Dementia Care Survey
MDQ: Moral Distress Questionnaire
MDT: Moral Distress Thermometer
MMD-HCP: Measure of Moral Distress for HCP
ProQOL: Professional Quality of Life Scale
RMSEA: Root mean squared error of approximation
SCQ: Stress Conscience Questionnaire
SRMR: Standardized root mean squared residuals
WLSMV: Weighted Least Square Mean and Variance adjusted estimator
