# Supplementary material for: Spanish psychometric properties of the moral distress scale—revised: a study in healthcare professionals treating COVID-19 patients
Source: BMC Med Ethics. 2023 May 12;24:30. doi: 10.1186/s12910-023-00911-2 (PMC10180620; doi:10.1186/s12910-023-00911-2)
Supplement: Supplementary file 1 — Supplementary Material 1: The Spanish version of the Moral Distress Scale [file 12910_2023_911_MOESM1_ESM.docx]

**Annex 1.** The Spanish version of the Moral Distress Scale – Revised.

Instrucciones: Lee las siguientes afirmaciones e indica del 0 (nunca) al 4 (siempre), con qué frecuencia se presentan o suceden eventos moralmente estresantes en un día de trabajo.

[*Instructions: Read the following statements and indicate from 0 (never) to 4 (always), how often morally stressful events occur or occur in a workday.*]

| **Items** | **0 – Nunca**  **[Never]** | **1** | **2** | **3** | **4 – Siempre**  **[Always]** |
| --- | --- | --- | --- | --- | --- |
| 1. Emprender acciones de reanimación cardiorrespiratoria cuando creo que solo retrasarán la muerte.  *[Original item number: 4*  *Initiate extensive life-saving actions when I think they only prolong death]* | 0 | 1 | 2 | 3 | 4 |
| 2. Prolongar cuidados a un paciente terminal mantenido con vida con ventilación mecánica, cuando nadie quiere tomar la decisión de retirar este soporte.  *[Original item number: 7*  *Continue to participate in care for a hopelessly ill person who is being sustained on a ventilator, when no one will make a decision to withdraw support.]* | 0 | 1 | 2 | 3 | 4 |
| 3. Asistir a un médico que, desde mi punto de vista, presta cuidados inadecuados.  *[Original item number: 9*  *Assist a physician who, in my opinion, is providing incompetent care.]* | 0 | 1 | 2 | 3 | 4 |
| 4. Ser obligado a prestar a pacientes un cuidado para el que no me siento cualificado.  *[Original item number: 10*  *Be required to care for patients I don’t feel qualified to care for.]* | 0 | 1 | 2 | 3 | 4 |
| 5. Presenciar casos en que estudiantes de medicina lleven a cabo procedimientos dolorosos en pacientes solo para mejorar sus competencias.  *[Original item number: 11*  *Witness medical students perform painful procedures on patients solely to increase their skill.]* | 0 | 1 | 2 | 3 | 4 |
| 6. Prestar cuidados que no alivian el sufrimiento del paciente porque el médico cree que aumentar la dosis de sedantes puede causar la muerte.  *[Original item number: 12*  *Provide care that does not relieve the patient’s suffering because the physician fears that increasing the dose of pain medication will cause death.]* | 0 | 1 | 2 | 3 | 4 |
| 7. No actuar frente a un dilema ético cuando un miembro del equipo implicado me pide no hacer nada.  *[Original item number: 15*  *Take no action about an observed ethical issue because the involved staff member or someone in a position of authority requested that I do nothing.]* | 0 | 1 | 2 | 3 | 4 |
| 8. Trabajar con enfermeras u otros profesionales de la salud, que no considero suficientemente cualificados para prestar los cuidados que requiere el paciente.  *[Original item number: 17*  *Work with nurses or other healthcare providers who are not as competent as the patient care requires.]* | 0 | 1 | 2 | 3 | 4 |
| 9. Obviar situaciones en las que los pacientes no han recibido la información apropiada para dar un consentimiento informado.  *[Original item number: 19*  *Ignore situations in which patients have not been given adequate information to insure informed consent.]* | 0 | 1 | 2 | 3 | 4 |
| 10. Constatar cuidados deficientes a un paciente por falta de medios para dar continuidad al tratamiento.  *[Original item number: 20*  *Watch patient care suffer because of a lack of provider continuity.]* | 0 | 1 | 2 | 3 | 4 |
| 11. Trabajar con un número de enfermeras u otros profesionales sanitarios que considero inseguro.  *[Original item number: 21*  *Work with levels of nurse or other care provider staffing that I consider unsafe.]* | 0 | 1 | 2 | 3 | 4 |
